# The Potential Anti-psoriatic Effects of Andrographolide: A Comparative Study to Topical Corticosteroids

**DOI:** 10.2174/0127722708296983240424102212

**Published:** 2024-05-06

**Authors:** Indira Dharmasamitha, Luh Made Mas Rusyati, Dyah Kanya Wati, I. Made Agus Gelgel Wirasuta

**Affiliations:** 1 Department of Dermatology, General Hospital Prof. Dr. I.G.N.G Ngoerah, Faculty of Medicine, Udayana University, Denpasar, Indonesia;; 2 Pediatric Consultant, Critical Care Medicine Udayana University, General Hospital Prof. Dr. I.G.N.G Ngoerah, Denpasar, Indonesia;; 3 Pharmacy Department, Faculty of Mathematics and Natural Science, Udayana University, Kuta Selatan, Indonesia

**Keywords:** Andrographolide, psoriasis, emulgel, sambiloto, *Andrographis paniculate*, anti-inflammatory, non-steroid treatment

## Abstract

***Background*:** Andrographolide (AP), a bioactive anti-inflammatory compound of Sambiloto, inhibits NF-κB, TNF-α, and interleukin IL-6. Nowadays, molecular docking simulation between AP and dexamethasone against NF-κB receptor presented the energy AP higher than dexamethasone. This becomes a potential treatment for psoriasis.

***Objective*:** This manuscript reported the effectiveness of AP from Sambiloto in treating psoriasis compared to topical steroids.

***Methods*:** This study conducted TLC analysis of AP content and its metabolite impurities, emulgel formulation, molecular docking, *in-silico* skin toxicity study, and *in-vivo* anti-psoriatic activity. This was a combination study of an *in-silico* study and an *in-vivo* study. This *in-silico* study was analyzed through multivariate statistical analysis (PCA) to elucidate the data constellation relationship of andrographolide derivatives with several target proteins. The intervention was performed in seven days. The PASI score, molecular parameters (IL-6, IL-17, VEGF, and TNF-a levels), and histopathological findings were assessed.

***Results*:** Molecular docking results revealed andrographolide to exhibit a relatively high binding affinity towards IL-6, NF-kB, and TNF-α which is comparable to the corticosteroids, andrographolide also shares similar residue interaction profile with each of the respective protein’s native ligand. In the *in-vivo* study, we found several parameters statistically significantly different regarding the intervention, including final PASI score (*p* = 0.017), redness (*p* = 0.017), scale (*p* = 0.040), thickness (*p* = 0.023), total histopathology of psoriasis score (*p* = 0.037), keratin layer score (*p* = 0.018).

***Conclusion*:** Emulgel AP 0.1% could lower the anti-inflammatory agent, which is vital to psoriasis progression.

## 1 INTRODUCTION

Andrographolide (AP), a bioactive anti-inflammatory compound of Sambiloto (*Andrographis paniculate*), inhibits NF-κB, TNF-α, and interleukin IL-6 [1]. The pro-inflammatory cytokines interleukin 23 (IL-23), IL-6, IL-1, and TNF-α are associated with the pathophysiology of psoriasis. Oral administration of AP at doses of 5 or 10 mg/kg mice dramatically decreased the mRNA expressions of IL-23 and IL-1β and prevented the emergence of skin autophagic proteolysis [2]. AP strongly inhibits human HaCaT keratinocytes so that it can be an anti-proliferative property [3]. Topical corticosteroid is the main therapy for treating inflammatory skin in psoriasis vulgaris. Long-term use of topical corticosteroids induces tachyphylaxis, skin atrophy, telangiectasia, striae, hirsutism, rebound flare phenomena, and adrenal suppression. Molecular docking simulation between AP and dexamethasone against NF-κB receptor presented the energy AP higher [4] than dexamethasone, which is based on presuming that AP has lower side effects compared to steroids. This becomes a potential treatment for psoriasis.

Around 92.6% of psoriasis vulgaris patients in Indonesia receive topical and systemic combination therapy. Meanwhile, the statistics abroad show that the use of topical preparations in the treatment of psoriasis reaches 80-90% [5]. This requires the development of AP as a topical preparation.

Molecular docking simulation is a computational method to predict interactions between two molecules, namely ligands and proteins. The scoring function evaluates conformations by calculating the strength of the affinity between the ligand and the protein and then directing the exploration of the ligand conformation to the pose with a stronger affinity. The difference in the strength affinity between the ligand and receptor can illustrate the strength of their potential pharmacological effect. Neoandrographolide (NAP) and Deoxyandrographolide (DAP) are derivatives of AP, all of which are secondary metabolites of *Sambiloto.* The topical preparation of AP, extracted from *Sambiloto,* is predominantly contaminated by NAP and DAP. Desoximetasone, hydrocortisone, mometasone, betamethasone dipropionate, and clobetasol propionate are corticosteroid active ingredients usually used for topical psoriasis treatment. The principal receptors of psoriasis treatment are NF-κB, TNF-α, and interleukin IL-6. The molecular docking simulation between those main psoriasis receptors again AP and its derivates and steroid active compounds produces a complex dataset. PCA (Principal Component Analysis) is a multivariate statistical technique used to reduce a datasets dimensionality and map or group variables suspected to be interrelated into the appropriate factors. With the help of PCA, the constellation between datasets of energy affinity bonding will be described and elaborated to predict the pharmacological potency effect of AP.

In this study, the potential of andrographolide in the treatment of psoriasis was developed. Research development includes molecular docking research to compare the pharmacological potency effect of andrographolide in psoriasis treatment compared to corticosteroids. It predicted topical toxicity that may appear when using andrographolide as a topical psoriasis treatment preparation compared to topical corticosteroids. The PCA constellation energy affinity bonding between ligands and psoriasis receptors can potentially be used for dose preparation of emulgel AP as a topical therapy for psoriasis. An *in-vivo* preclinical study on psoriasis animal test models was conducted to test the activity of emulgel AP on psoriasis treatment.

This manuscript reported AP extraction from Sambiloto, TLC analysis of AP content and its metabolite impurities, emulgel formulation, molecular docking, *in-silico* skin toxicity study, and *in-vivo* anti-psoriatic activity.

## 2 MATERIALS AND METHODS

### 2.1 Molecular Target

This *in-silico* study identified several target proteins, including IL-6, NF-kB, and TNF-α. Optimization of the three-dimensional structures of targeted ligands (andrographolide compounds, deoxyandrographolide, neoandrographolide, desoximetasone, hydrocortisone, and mometasone) was used for the preparation of target proteins was carried out, followed by the validation of the molecular docking method. The crystal structure of the protein target and the 3D structure of ligands were downloaded from the protein data bank and retrieved from the PubChem database (https://pubchem.ncbi.nlm.nih.gov/). These structures have been subsequently optimized by using the HyperChem 8 software. Protein Data Bank obtained the three-dimensional structural database of the target protein (http://www.rcsb.org/pdb/home/home.do), which will be further processed using the Chimera 1.11 software. Auto dock tools were used to find the Gibbs free energy between ligand and target proteins. The bond energy and the nature of the bond formed between the chemical and the target protein are the key outcomes of molecular docking. The potential types of bonds include hydrogen, Van der Waals, and electrostatic bonds. The identification of the binding pocket is the prerequisite for site-specific docking. After the binding pocket analysis, it was suggested that the amino acids, *e.g.*, ARG179, ARG182, PHE535, LEU472, TYR151, GLN175, ARG408, and SER410 are present in the active binding pocket targeted proteins. The outcomes of *in-silico* simulations were systematically tabulated using Auto dock between the ligand and each receptor. The energy bonding between ligands and receptors was tabulated. The constellation between datasets of energy affinity bonding will be described and elaborated to predict the pharmacological potency effect of AP for PCA analysis. The results of *in-silico* docking will be used to determine the dose of emulgel concentration.

### 2.2 Extract Preparation

AP was extracted from 1 kg of sambiloto plant, according to Warditiani, 2020. TLC conducted impurities of derivates AP. AP content on Sambiloto’s extract was determined by TLC spectrophotodencitometry [6]. Formulation of emulgel was used patent formula No S00202203885.

### 2.3 *In-vivo* Study Design and Sample Grouping

This was an experimental study with a randomized post-test-only control group design. The minimum sample was 19 rats. The minimum sample size was obtained according to the Frederer sample calculation formula. All of the samples were collected using simple random sampling techniques. We obtained the animals from the laboratory staff at the Histology Department, Faculty of Medicine, Udayana University, who are experts in handling animal research studies. We have to complete the order form which consists of order date, animal requirement and cost. The sample was available 1 month after the order, waiting for samples to meet the specifications. The studys inclusion criteria were rat *Rattus novergicus* strain *Wistar*, male, weight 300-400 grams, and around 3-4 months. On the other hand, the exclusion criteria in this study were defective or dead during the study. In this study, there were no samples that were excluded. Before the research began, the authors determined the study design and procedures, but these were not registered.

The sample was classified into seven groups. Group I was a normal group that only got a placebo gel and consisted of 2 rats. The control group was divided into negative and positive control groups. Group II (IMQ control) was a negative control group. The sample was applied with IMQ (Aldara cream w/w) in this group to induce psoriasis. After 4 hours, a placebo gel was applied. Group III (IMQ and Desoxymethasone) was a positive control group that used the topical Imiquimod 5% 62.5 mg on the back of rats per day. After 4 hours, we applied the 0.25% desoxymethasone cream. Group IV (IMQ and Momethasone) was a positive control group that used the topical Imiquimod 5% 62.5 mg on the back of a rat per day. After 4 hours, 0.1% mometasone cream was applied to the sample. In this study, the interventional group was divided into three groups. Each group will be given a topical Imiquimod 5% 62.5 mg on the back of the rat daily. After 4 hours, all samples will be applied topical andrographolide in different concentrations, such as 0,1% (group V/A1), 0.175% (group VI/A2), and 0.25% (group VII/A3). To minimize the treatment errors, all samples were grouped according to the intervention given and then placed in labeled cages. The intervention was performed in 7 days. The observation was extended by two days to observe the inflammatory process in psoriasis model rats. All of the authors are aware of the group allocation at the different stages of the experiment.

### 2.4 *In-vivo* Research Procedure

The rats were acclimatized for seven days to reduce stress before the beginning of the study. The rats were shaved around 500 mm^2^ in the dorsal area or below near the tail. Documentation of the skin was performed before applying topical IMQ. The dose of each rat was a quarter of a cassette of topical IMQ to induce psoriasis. Thus, up to 4 ¾ cassettes were used for 17 rats in a day. Application of a quarter of a cassette of IMQ was usually at 10:00 a.m. Four hours after the IMQ application, each group was treated according to their type and treatment concentration. A skin biopsy with an area of 1 cm was performed on the ninth day. Meanwhile, the tissue samples were fixed in a 10% formalin buffer. All tissues were placed in paraffin blocks and cut with a 4-5 µM thickness. Each section was deparaffinized with xylene, serially scaled with alcohol to water, and then stained with hematoxylin-eosin (HE) for standard evaluation with an Olympus CV microscope. Immunohistochemical examination then continued to assess the expression of proinflammatory cytokines and angiogenesis. In addition, this study also measured the PASI score and molecular parameters, including IL-6, IL-17, VEGF, and TNF-a levels. The molecular parameters were examined by using an ELISA kit. There were no unexpected adverse events during the study.

### 2.5 *In-vivo* Statistical Analysis

In this study, the data was presented descriptively and analytically. The descriptive research was presented in graphics and a table. The analytical study used in this study was the Kruskal-Wallis and Dunn test for post hoc analysis.

## 3 RESULTS

### 3.1 *In-silico* Molecular Docking and PCA Analysis Study

**Fig. (1):**
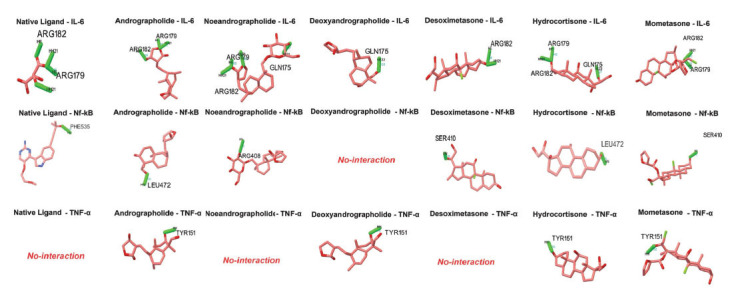
Hydrogen bond interaction with its respective ligands.

According to the molecular docking results, hydrocortisone, mometasone, and neoandrographolide exhibited the highest binding affinity towards IL-6, in descending order. Conversely, native ligands showed the highest binding affinity towards NF-kB, followed by two corticosteroids (desoximetasone and clobetasol propionate). In the final docking towards TNF-α, betamethasone, hydrocortisone, and neoandrographolide demonstrated the highest binding affinity towards TNF-α (Table **1**). Interestingly, two ligands, the native ligand, and neoandrographolide, formed four hydrogen bond interactions with IL-6, more than any of the corticosteroids. In contrast, all compounds and corticosteroids formed only one hydrogen bond interaction with NF-kB. The interaction with TNF-α was similar to NF-kB, with an average of one hydrogen bond formed, except for its native ligand. It was observed that andrographolide also shared similar residue interactions (ARG 179 ARG 182) when comparing the residues responsible for forming hydrogen bonds between the native ligand and IL-6. Interacting with similar residues could indicate a similar molecular effect of the native ligand on IL-6. Neoandrographolide also interacted with residues like the native ligand when paired with IL-6. This observation is further supported by the fact that all the corticosteroids share the same residue interaction of the native ligand with IL-6, which includes either residue ARG 179 or ARG 182, and in some cases, both (Table **2** and Fig. **1**).

**Fig. (2):**
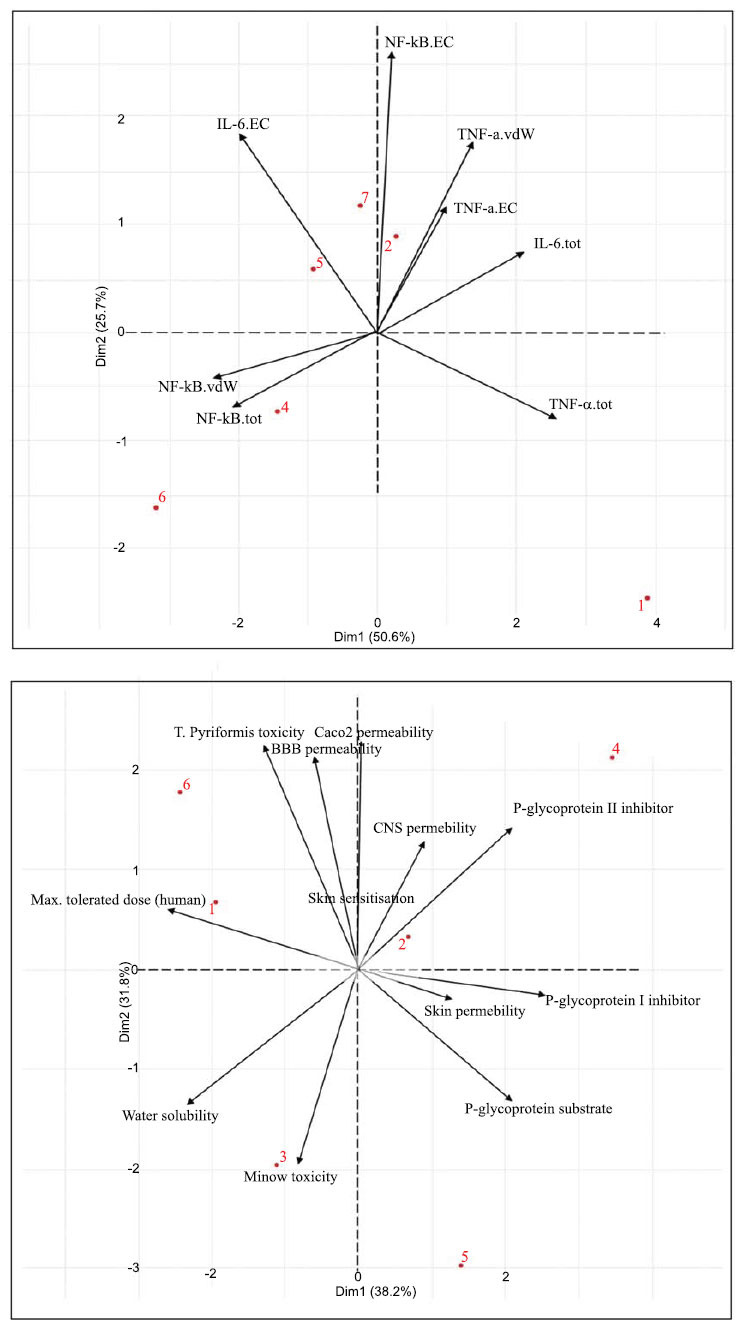
PCA analysis biplot.

A multivariate analysis of the properties of andrographolide variants alongside corticosteroids was also conducted. Based on the results of the biplot (Fig. **2**, left), it provides a comprehensive representation of individual data and variables, encompassing a total of six observations of individual data (1 = Andrographolide, 2 = Desoximetasone, 3 = Hydrocortisone, 4 = Mometasone, 5 = Neoandrographolide, 6 = Deoxyandrographolide). Most variables are well represented within the two dimensions created in this Principal Component Analysis, except for skin sensitization, CNS permeability, and skin permeability. Certain variables exhibit inverse relationships, such as max tolerated dose in humans and water solubility against P-glycoprotein I inhibitor, P-glycoprotein II inhibitor, and P-glycoprotein substrate. Conversely, other variables demonstrate linear relationships, such as the interaction between CaCO_2_, blood-brain barrier, and *T. piriformis* toxicity. However, the inverse interaction is more pronounced than the linear relationships, as indicated by the variables creating an angle approaching 180 degrees. The interaction between individual data and the variable vector shows that andrographolide exhibits the highest max tolerated dose in humans, as indicated by its proximity to the maximum dose variable. Similarly, deoxyandrographolide, while also close to the max dose variable, indicating a relatively high max dosage in humans, is notably close to the *T. piriformis* toxicity variable, suggesting a relatively high toxicity. Neoandrographolide, on the other hand, is closer to the minnow toxicity variable, albeit not as close as hydrocortisone-Mometasone and neoandrographolide show an affinity towards the P-glycoprotein II inhibitor variable.

The second biplot (Fig. **2**, right) demonstrates a relatively robust representation of all variables within the principal component, with TNF- α being the sole exception. As the similar vector direction indicates, variables such as TNF- α.tot and IL-6.vdW appear to be highly positively correlated. However, TNF- α.tot does not correlate strongly with its counterparts TNF- α.vdW and TNF- α.EC. IL-6.tot and NF-kB.tot appear highly negatively correlated, as suggested by the angle approaching 180 degrees. NF-kB.tot seems to be influenced by its Van der Waals variable counterpart, while IL-6 appears minimally affected by IL-6.EC and IL-6.vd. Regarding the individual data, the biplot observes seven individual data points (1 = Native Ligand, 2 = Andrographolide, 3 = Desoximetasone, 4 = Hydrocortisone, 5 = Mometasone, 6 = Neoandrographolide, 7 = Deoxyandrographolide). Deoxyandrographolide and andrographolide appear to influence NF-kB.EC. Both are also relatively close to TNF- α.vdW and TNF- α. Desoximetasone’s impact on both variables overshadows EC, but their influence. Neoandrographolide, on the other hand, heavily influences NF-kB.vdW and NF-kB.tot, with hydrocortisone also showing some influence, albeit to a lesser extent.

**Fig. (3):**
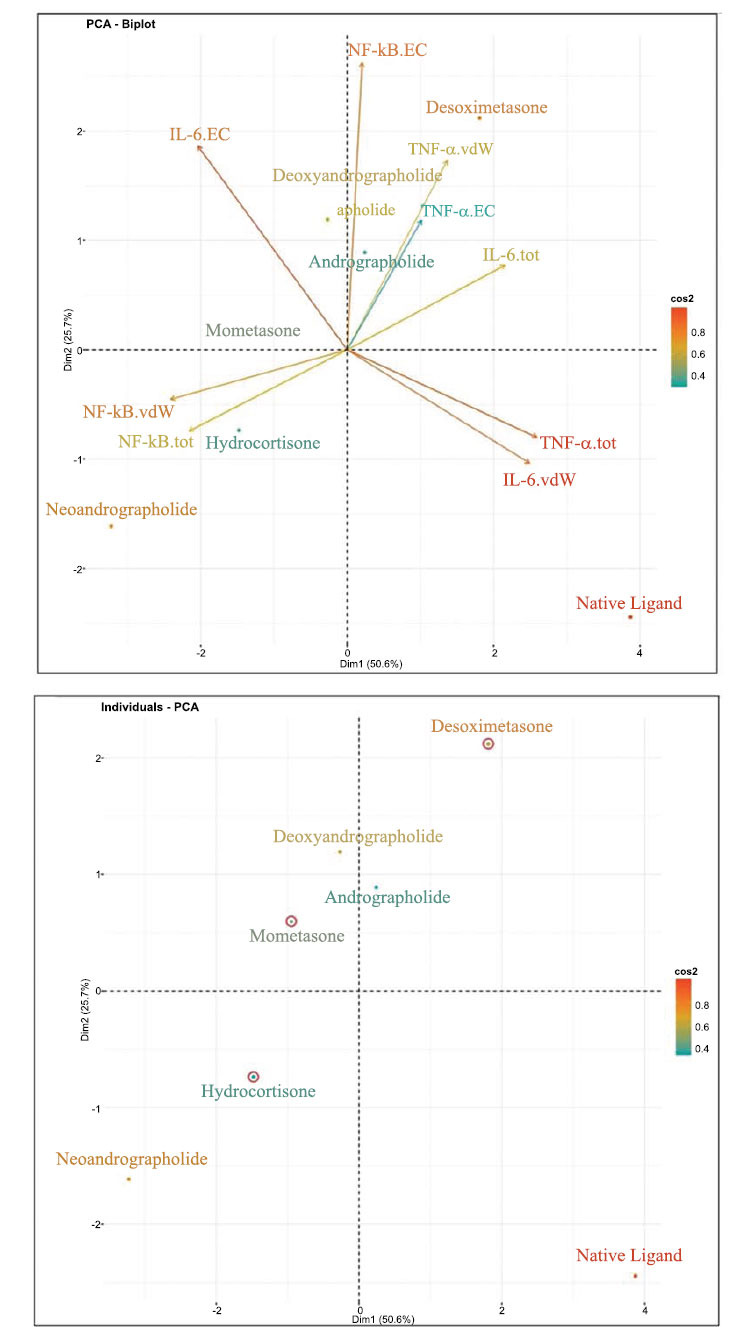
Individual data PCA.

Aside from biplots from the PCA analysis, PCA correlation (Tables **3** and **4**) and individual plots (Fig. **3**) were also produced. PCA correlation revealed that the first dimension is significantly influenced by five variables when the cutoff is set to 0.5. These variables include water solubility, P-glycoprotein substrate, P-glycoprotein I inhibitor, P-glycoprotein II inhibitor, and max tolerated dose in humans. Water solubility and max tolerated dose correlate negatively or inverse with the first dimension, while the three P-glycoprotein-related variables correlate positively. Like the first dimension, water solubility and P-glycoprotein II inhibitors also heavily influence the second dimension. Additional variables such as CaCO_2_ permeability, blood-brain barrier permeability, and *T. piriformis* toxicity strongly influence the second dimension. The second PCA correlation results (Table **4**) demonstrate a high correlation between the first dimension and almost all variables   except   the   NF-kB-EC variable. Three variables (IL-6.EC, NF-kB.tot, NF-kB.vdW) exhibit an inverse correlation, while the others correlate linearly with the first dimension. In contrast to the first dimension, the second dimension correlates with fewer variables, with only TNF-α.vdW, NF-kB.EC, and IL-6.EC showed a high correlation with the second dimension. Finally, the individual data PCA plot (Fig. **3**) revealed deoxy andrographolide and andrographolide positioned between mometasone and desoximetasone. Given that data points clustered together to share similar properties, one of which is dosage, we can estimate the dosage used within deoxy andrographolide and andrographolide about mometasone 0.1% and desoximetasone 0.25%. Consequently, andrographolide should be between the dosage of mometasone 0.1% and desoximetasone 0.25%.

### 3.2 *In-vivo* Study

#### 3.2.1 Pro-inflammatory Cytokine Production

Several parameters pivotal inflammatory factors in psoriasis have been observed in this study, including IL-6, IL-17, TNF-α, and VEGF. The highest level of inflammatory factors level (IL-6, IL-17, TNF-α, and VEGF) was found in group II (6.83 ± 5.50 pg/ml, 15.16 ± 12.16 pg/ml, 13.83 ± 10.16 pg/ml, and 6.00 ± 4.33 pg/ml, respectively) (Table **5**). Besides group I, which did not get IMQ exposure, the lowest level of the parameters in each parameter was in group IV, followed by group V. Unfortunately, we found no significant differences among groups in all four parameters (*p* 0.05). The visualization of IL-6, IL1, TNF-α, and VEGF mean levels was reported in Fig. (**4**).

#### 3.2.2 PASI Score and Histopathology Evaluation

During the intervention, the PASI score in each group was evaluated every couple of days. Regarding the intervention, it showed that group VII had an enhancement of PASI score from day 3 until day 9. It meant the AP dosage in group VII was not appropriate. Regarding our study, we found that only group V had a decrease in PASI score from day 5 until day 9. Unfortunately, the group III and IV had fluctuating PASI scores. The PASI score in those groups decreased on day seven and increased on day 9. The stagnant PASI score was reported in groups VI and VII. Regarding our findings, we can conclude that group V (IMQ+AP 0.1%) had better outcomes rather than other intervention groups. The detailed data has been established in Fig. (**5**).

**Fig. (6):**
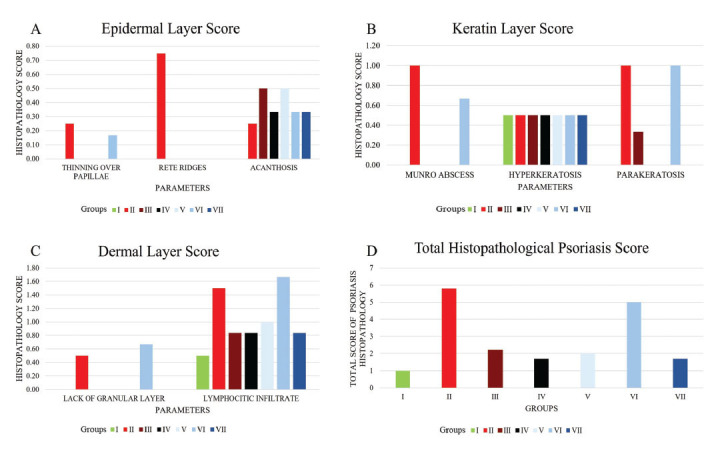
The histopathology score of psoriasis. **A**) Epidermal layer, **B**) Keratin layer, **C**) Dermal layer, **D**) Total histopathology of psoriasis. Group I (normal), group II (IMQ), group III (IMQ+desoxymethasone), group IV (IMQ+momethason), group V (IMQ+AP1%), group VI (IMQ+AP 0.175%), group VII (IMQ+ AP 0.25%).

A histology examination has been performed in this study. We found some groups had acanthosis, with the highest group being in groups III and V. Both rete ridges and thinning of the papillae were seen in only group II. Hyperkeratosis was the most frequent pathological process observed on the keratin layer. Several groups also found a munro abscess (groups II and VI) and parakeratosis (groups II, III and VI). Lymphocytic infiltrate was the most common finding in the dermal layer. The highest total histopathology of psoriasis score regarding the histology examination was found in group VI, followed by group II, group III, group V, group VII, and group I. According to the histological examination of the three layers of skin, all components related to the progressiveness of psoriasis were found in group II, such as acanthosis, parakeratosis, hyperkeratosis, lymphocytic infiltrates, *etc*. Interestingly, some histological abnormalities were still found in each intervention group. This indicates that the intervention given cannot produce the same outcome as group I (normal) in 9 days (Fig. **6**).

Our study observed several parameters for evaluating the progression of psoriasis, such as final PASI score, redness, scales, and thickness. The highest final PASI score was in the group II (6.50 ± 2.121), followed by the group VII (3.67 ± 0.577), group VI (2.67 ± 0.577), group III (2.33 ± 0.577), and the last were IV and V group (1.67 ± 0.577). This study also found a significant difference in the final PASI score with each group intervention (*p* = 0.017). We also evaluated each parameter of the PASI score, including redness, scale, and thickness. Group II became the group with the highest score of redness (2.50 ± 0.707), scale (1.50 ± 0.707), and thickness (2.50 ± 0.707) parameter. In line with the final PASI score, group V had the lowest score of scale thickness, and redness, compared to the group that received corticosteroid intervention. In this study, we also found a significant difference in the final PASI score and each component with the treatment intervention in each group (*p* 0.05) (Table **6**).

Histopathological evaluation found that the highest score was in group II (5.75 ± 4.59), followed by group VI (5.00 ± 2.29), group III (2.16 ± 0.76), group V (2.00 ± 0.00), group VII (1.67 ± 0.57), group IV (1.67 ± 0.28), and group I (1.00 ± 0.00). The differences were statistically significant (*p* = 0.037). The higher score in the keratin, epidermis, and dermis layers meant that the subject had a higher psoriasis histopathological score, resulting in a worse condition. The highest score of keratin and epidermal layer was in group II (2.50 ± 1.41 and 1.25 ± 1.76 respectively). Meanwhile, the highest dermal layer score was found in group VI (2.33 ± 1.15). No significant difference was observed in the epidermal and dermal layer (*p* = 0.634 and *p* = 0.103, respectively). In contrast, a significant difference was found in the keratin layer score (*p* = 0.018) (Table **6**).

**Fig. (7):**
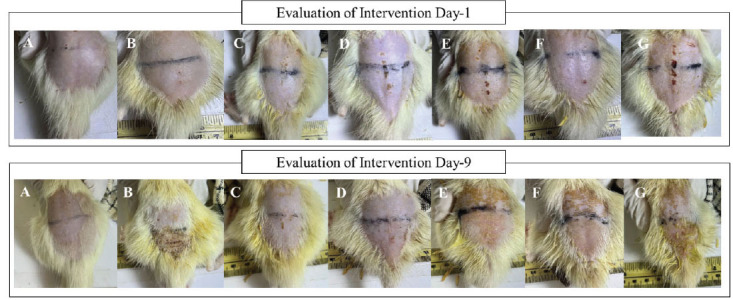
Rat model psoriasis in nine days evaluation. It reported the intervention comparison on day 1 and day 9. **A**) Normal group (Group I), **B**) IMQ group (Group II), **C**) IMQ and Desoxymethasone (Group III), **D**) IMQ and Momethasone (Group IV), **E**) IMQ and 0.1% AP (Group V), **F**) IMQ and AP 0.175% (Group VI), and **G**) IMQ and AP 0.25% (Group VII).

A multiple comparison was done to know the interaction between groups. According to this study, we found a significant difference in comparison group analysis of final PASI score between groups I and II (*p* = 0.002), I and VI (*p* = 0.041), groups I and VII (*p* = 0.005), II and IV (*p* = 0.016), II and V (*p* = 0.016), III and IV (*p* = 0.016), IV and VII (*p* = 0.043). The significant finding of groups II and V indicated that AP 0.1% has the potency to treat psoriasis condition by lowering the inflammation factors. However, there was a significant mean difference between groups IV (1.67 ± 0.577) and VII (3.67 ± 0.577) in the final PASI score. This condition indicates that group IV intervention is significantly better used in treating psoriasis than group VII due to the low final PASI score of group IV rather than group VII. We also found a significant difference in comparing groups IV and VII (*p* = 0.024) in scale parameters, which indicated that corticosteroids are better in treating rats model psoriasis due to a lower final PASI score rather than AP 0.25%. Treating the rat model psoriasis with AP 0.1% (Group V), reducing the thickness of the skin was better than group IV, which used corticosteroids. It can be seen by the significant difference in p-value between group II and V (*p* = 0.013) rather than group II and IV (*p* = 0.055) (Tables **7** and **8**). We did not find a significant difference in lowering the skin thickness in the treatment intervention beside groups IV and VII (*p* = 0.013) (Table **9**). Unfortunately, group IV was better at lowering the skin thickness rather than group VII. In contrast, all of the intervention groups had statistically significant differences in lowering the skin redness compared to group II (*p* 0.05) (Table **10**). In addition, groups IV and VI had statistically significant differences according to the psoriasis score (Table **11**). Group IV had the lowest psoriasis score compared to group VI. It indicated that corticosteroid was better in reducing the histological psoriasis parameter rather than AP 0.25%. A Similar finding was also found in the keratin score (Table **12**). In this study, we also presented the clinical features of rats before (day-1) and after (day-9) intervention (Fig. **7**). The histology examination is shown in Fig. (**8**).

## 4 DISCUSSION

Psoriasis is a chronic inflammatory skin condition that can cause excessive epidermal growth [7-9]. This condition is caused by increased keratinocyte proliferation, altered keratinocyte differentiation, and inflammatory cell infiltrates in the epidermis and dermis [10-12]. Most inflammatory infiltrates in the dermis comprise T lymphocytes, macrophages, neutrophils, and dendritic cells (DCs) [13]. DCs in the skin release proinflammatory cytokines such as tumor necrosis factor-alpha (TNF-α), interleukin 23 (IL-23), IL-6, and IL-1b as psoriasis spreads [14, 15]. The polarization of naive T cells to Th17 cells by IL-23 is associated with the proliferation of keratinocytes and other defining characteristics of psoriasis [16-19].

TNF-α had a pivotal role in psoriasis disease progression [20-22] The higher level of TNF-α could be associated with worse disease than the patient with a lower TNF-α level. Regarding our study, the psoriasis rat group II had a higher TNF-α level than groups V, VI, and VII, which got anti-inflammatory treatment although not significantly different (*p* 0.05). Therefore, lowering the TNF-α level is essential. A study by Gupta *et al.* found that anti-inflammatory treatment could reduce the TNF-α level in mice model rheumatoid arthritis disease. They discovered that Andrographolide 98% is better than dexamethasone at suppressing TNF-α. Other inflammatory molecules such as COX-2, NF-B, CD40, IL-1, and IL-6 were also low [23]. Various proinflammatory cytokines are involved, including IL-23 and IL-1b, which are produced in a way that is dependent on MyD88 [24]. A cytokine called IL-23, mainly released, can stimulate the growth of Th17 cells and the appearance of psoriasis-like symptoms [25]. The development of psoriasis is significantly influenced by the IL-23/Th17 axis and IL-1b [26-29].

In the disease progression of psoriasis, keratinocytes, immune cells, and endothelial cells are the targets of IL-17 [30]. In our study, group II had IL-17 levels of 15.16 ± 12.16 pg/mL, higher than the andrographolide-treated groups (group V at 3.00 ± 1.45 pg/ml; group VI at 3.89 ± 3.56 pg/ml; and group VII at 2.44 ± 1.07 pg/ml). Although not statistically significant, the high levels of IL-17 in group II indicate the role of IL-17 in the pathogenesis of psoriasis and the critical role of anti-inflammatory therapy in reducing IL-17 levels observed in groups three to seven. IL-17 also has a role in psoriasis comorbidities. A study by Egeberg *et al.* found the role of IL-17 on vascular dysfunction in psoriasis patients [31]. Studies by Karbach *et al.* and Schüler *et al.* reported that increased IL-17A expression was in line with increased keratinocyte thickening common in psoriasis patients, endothelial dysfunction, and arterial hypertension [32, 33]. Therefore, we can conclude the critical role of IL-17 in the disease progression of psoriasis.

The pathogenesis of psoriasis is complicated, and the therapy is still a major challenge [16, 34-37]. Topical corticosteroid cream has been the primary treatment for inflamed skin in psoriasis vulgaris until now. Through an anti-inflammatory, antiproliferative, immunosuppressive, and vasoconstriction mechanism of action, topical corticosteroids combat psoriasis. However, extended corticosteroid treatment must be constantly monitored because it may produce symptoms like rebound flares, striae, telangiectasia, tachyphylaxis, skin atrophy, and adrenal suppression [1]. Observing the gap in existing psoriasis treatments, natural compounds such as andrographolide can overcome the shortcomings of these treatments.

Andrographolide is a small molecule compound derived from Andrographis (*Andrographis paniculata*), a Chinese herbal plant [38-41]. In China, India, Japan, and Korea, Andrographis has a long history of use in treating inflammatory diseases. It is currently used in China to treat rheumatoid arthritis, diarrhea, and laryngitis [42, 43]. A study conducted by Shao *et al.* (2016), which tested andrographolide compounds in imiquimod-induced psoriasis mice, showed that the andrographolide can reduce imiquimod- but not IL-23-induced psoriasis in mice. Mice lacking the MAP1LC3B protein did not exhibit the improvement in imiquimod-induced psoriasis brought on by the andrographolide compound. Additionally, andrographolide reduced IL-23, IL-6, and IL-1b mRNA expressions in bone marrow-derived dendritic cells treated with lipopolysaccharide. Additionally, andrographolide reduced the IL-23, IL-6, IL-1b, CD80, and CD86 mRNA expressions stimulated by imiquimod in mouse BMDCs [2].

The other pathogenetic mechanism in psoriasis is represented by VEGF-induced angiogenesis. Psoriasis patients have high serum levels of VEGF [44-47] and endothelial cell stimulating angiogenesis factor (ESAF), and the severity of psoriasis correlates with serum VEGF levels. This is attributed to the abnormal function of the epidermal layer as a skin barrier. Increased VEGF levels are in line with increased keratinocyte hyperplasia. For this reason, it is concluded that the process of keratinocyte cell proliferation is supported by VEGF, as seen from the increase in VEGF levels in psoriasis patients [47]. This mechanism begins when the VEGF receptors bind with the ligand. The results express the VEGF factor and different types of cells, such as monocytes/macrophages and keratinocytes. Those factors are closely related to the pathogenesis of psoriatic disease [48]. Regarding our study, we found that group II had VEGF levels of 6.00 ± 4.33 pg/mL, higher than the andrographolide-treated groups (group V of 1.11 ± 1.07 pg/ml; group VI of 2.11 ± 2.49 pg/ml; and group VII of 1.11 ± 0.50 pg/ml). Although VEGF levels were lower in the andrographolide-treated group, the difference was insignificant (*p* 0.05).

An *in-silico* analysis was also performed to validate previous *in vivo* findings further. Our results established that the overall interaction between andrographolide and the target proteins was weaker than the corticosteroids. Its interaction with IL-6 (5.92 Kkal/mol) was relatively stronger than most of the corticosteroids mentioned above, except hydrocortisone (-6.97 Kkal/mol) and mometasone (-6.5 Kkal/mol). Interestingly, one of the variants of andrographolide, neo-andrographolide, exhibited better binding affinity than andrographolide when interacting with IL-6 (-6.43 Kkal/mol). This finding is similar to other studies that depict similar capabilities of andrographolide in binding and inhibiting IL-6 [39, 49-52]. However, when interacting with NF-kB, the binding affinity of andrographolide was overshadowed by most of the corticosteroids, except for betamethasone dipropionate, which showed positive binding affinity indicating a non-spontaneous interaction (-6.26 Kkal/mol *vs* 11.74 Kkal/mol, -7.63 Kkal/mol, -8.18 Kkal/mol, -9.53 Kkal/mol, -9.66 Kkal/mol). Finally, interactions with TNF-α showed minor deviation in binding affinity across andrographolide variants and the corticosteroids, with neoandrographolide (-6.87 Kkal/mol), hydrocortisone (-6.92 Kkal/mol), and betamethasone (-7.1 Kkal/mol) demonstrating the highest binding affinity, this indicates andrographolide’s ability to interact with TNF-α is on par with corticosteroids which is further validated by another study by Tarachnand *et al.* (2023) [53]. Overall, the difference in binding affinity of andrographolide and its variants compared to its corticosteroid counterparts did not show a lot of deviation, which might indicate similar properties to that of the corticosteroids. Some hydrogen bonds were formed when the andrographolide compounds and the corticosteroids interacted with the target protein. Two of all the ligands created four hydrogen bond interactions towards IL-6, its native ligand, and neoandrographolide. This was more hydrogen bonds than all of the corticosteroids, which might indicate a more stable interaction with IL-6. In contrast to IL-6, all compounds, and corticosteroids only made one hydrogen bond interaction with NF-kB. Lastly, TNF-α hydrogen bond interaction with the compounds and corticosteroids was similar to NF-kB, where, on average, it only creates one hydrogen bond except its native ligand. From its hydrogen bond interaction profile, it would appear that the andrographolides share a similar, if not a more stable interaction, to the corticosteroids. This further insinuates that the exchange of andrographolides may resemble that of the corticosteroids, resulting in similar, if not better, effects on psoriasis [54, 55].

Further *in-silico* investigation of andrographolide revealed its potential to prevent atherosclerosis [50, 56-58]. The study found a molecular binding between the extract of *Andrographis paniculata*, commonly known as sambiloto, and atherosclerosis target proteins. The optimal affinity energy bonds (kcal/ mol) between sambiloto extract and target proteins (NF-kB, ICAM-1, VCAM-1, TNF-α, IFN-γ, Cyt MAP kinase P32) were -7.9, -7.3, -6.5, -7.8, -6.2, and -7.7, respectively. In contrast, the optimal affinity energy bonds (kcal/ mol) between the reference substance and target proteins were -8.5, -7.9, -7.2, -8.5, -6.9, and -7.6, respectively. The energy bonds of dexamethasone with NF-kB, ICAM-1, VCAM-1, IFN-γ, and TNF-α were more negative than those with sambiloto extract. Interestingly, dexamethasone bond energy on cytokine MAP kinase P32 was even more damaging than the sambiloto extraction [59-62]. The bond energy values of sambiloto extract with those target proteins were not significantly different from dexamethasone. These results suggest that the sambiloto section binds to target proteins and inhibits atherosclerosis production, demonstrating its potential therapeutic efficacy.

Clinically, one measure of psoriasis improvement is a decline in PASI score [63]. Psoriasis symptoms differ from person to person and might worsen as people age. The highest final PASI score was in the group II (6.50 ± 2.121), followed by the group VII (3.67 ± 0.577), group VI (2.67 ± 0.577), group III (2.33 ± 0.577), and the last were IV and V group (1.67 ± 0.577). This study also found a significant difference in the final PASI score with each group intervention (*p* = 0.017). We found a significant difference in comparison group analysis of final PASI score between groups I and II (*p* = 0.002), I and VI (*p* = 0.041), groups I and VII (*p* = 0.005), II and IV (*p* = 0.016), II and V (*p* = 0.016), III and IV (*p* = 0.016), IV and VII (*p* = 0.043). The significant finding of groups II and V indicated that AP 0.1% can treat psoriasis by lowering the inflammation factors. However, there was a significant mean difference between groups IV (1.67 ± 0.577) and VII (3.67 ± 0.577) in the final PASI score. This condition indicates that group IV intervention is significantly better used in treating psoriasis than group VII due to the low final PASI score of group IV rather than group VII. These results show that andrographolide could potentially treat psoriasis by evaluating the PASI score in rat model psoriasis.

In addition, Shao *et al.* (2016), who examined dorsal skin thickness from andrographolide-administered experimental animals, also showed that andrographolide significantly inhibited pathological changes in keratinocytes dose-dependently. Andrographolide also ameliorated inflammation and scaling of psoriasis in the skin of mice treated with IMQ. They found that a dose of andrographolide at 10 mg/kg was as effective as etanercept at 10 mg/kg, one of the treatment modalities for psoriasis [2]. Despite using different parameters, both studies concluded that andrographolide has potential for psoriasis patients.

This study found that topical andrographolide with higher concentrations (0.175% and 0.25%) could not reduce the PASI score effectively. This could be due to the side effects of high concentrations, which can be irritative, as we predicted during molecular docking. Indeed, to date, our study is the first to use andrographolide extract topically, while previous studies used the extract orally and by *in vitro* study (using HaCaT cell line) [2, 3, 64, 65]. To the best of our knowledge, our study is the first to correlate the effect of andrographolide on PASI score. However, in other clinical variables such as skin redness, quantity of scales formed, and gross thickness of the skin, there was no significant difference between all groups (*p* 0.05).

Psoriasis is a dynamic dermatosis with morphological changes during the evolution of an individual lesion [66, 67]. In the initial stage, due to the vasodilatation process, it leads to lymphocytic infiltration and edema. After that, the epidermis loses the granular layer and thickens. In this phase, keratinocytes proliferate and mature rapidly, leading to incomplete terminal differentiation. The existence of scale and flake lesions characterizes this condition. We also found parakeratosis and munro abscess. In the advanced stage, acanthosis, rete ridges elongation, and suprapapillary thinning exist. Moreover, due to transmigration, the inflammation cell on the parakeratotic scale in the epidermis also causes the formation of a Munro abscess. The inflammation levels are higher in this stage than in the initial step. Thus, we can find the Langerhans cell, T lymphocyte, and CD11c. T-lymphocytes are also found in the keratin layer. In the later lesion, we can find an orthokeratosis [67]. Regarding our study, we found that every sign in every layer was found in group II, indicating that group II was in the advanced phase. The group treated with corticosteroids (Group III and IV) found only acanthosis in the epidermal layer, hyper parakeratosis, parakeratosis (group III only) in the keratin layer, and lymphocytic infiltrate. These findings also indicated that both groups were in the advanced phase. However, we only found an acanthosis as a sign of the advanced stage. This finding was also quite similar to the andrographolide group. This means both interventions could reduce the inflammation cells in rats. Regarding the total histopathology of psoriasis score, group IV (2.00 ± 0.00) had the lowest score, after that followed by group VII (1.67 ± 0.57), V (2.00 ± 0.00), III (2.16 ± 0.76), and VI (5.00 ± 2.29). This finding is statistically significant (*p* = 0.037). Interestingly, the total histopathology of psoriasis score was not in line with the final PASI score. We found an increase in PASI score in the AP group, which was in line with the increase in AP dose. In contrast with the Kim *et al.* study, most histologic findings showed significant change as the gross lesion size increased (*p* = 0.05). However, the PSI score showed no statistical correlation with histopathological severity [68]. This finding indicates that histopathology is not in line with the gross lesion (a.g PASI score) due to several factors that may impact the gross lesion.

Histopathology as an objective examination of the psoriasis severity index revealed a significant difference in the keratin layer with the intervention, in which groups IV, V, and VII had the lowest scores. Unfortunately, we could not find a significant difference between epidermal layer and dermal layer scores for the intervention (*p* = 0.634 and 0.103, respectively). Therefore, we can conclude that the corticosteroid or AP intervention had a similar effect on psoriasis by determining the mean differences that are not significantly different. Conversely, a meta-analysis found a significant association of epidermal thickness with the curcumin intervention (*p* = 0.01) [69].

This study certainly has limitations in that we only included a few animals. Therefore, it may affect the mean and standard deviations of every variable observed in this study. A larger study with more animals is needed to confirm the findings of this study. Further research, such as clinical trial research, is necessary to determine the efficacy of andrographolide.

## CONCLUSION

In conclusion, andrographolide extract can decrease anti-inflammatory agents that have an essential role in developing psoriasis. Moreover, various results were found for each parameter. Based on several AP concentrations, AP 0.1% has potential as a herbal therapy and can be an option for psoriasis compared to other AP concentrations. Nonetheless, further studies are needed to confirm the results we found in this study.

## AUTHORS' CONTRIBUTIONS

 Study conception and design: ID and IMAGW. Data collection: LMMR and DKW. Analysis and interpretation of results: ID, and IMAGW. Preparing the draft manuscript: ID, LMMR and DKW. All authors reviewed the results and approved the final version of the manuscript.

## Figures and Tables

**Fig. (4):**
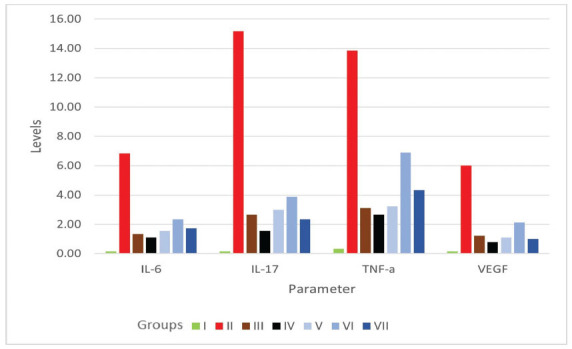
Differences in IL-6, IL-17, TNF-α, and VEGF levels among groups. Group I (normal), group II (IMQ), group III (IMQ+desoxymethasone), group IV (IMQ+momethason), group V (IMQ+AP1%), group VI (IMQ+AP 0.175%), group VII (IMQ+ AP 0.25%).

**Fig. (5):**
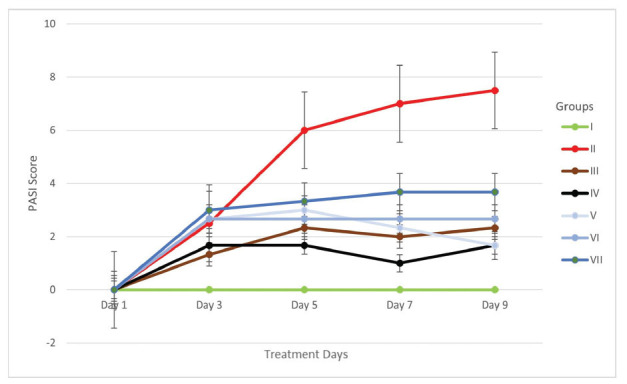
The progression of the PASI score in each group in nine days. Group I (normal), group II (IMQ), group III (IMQ+desoxymethasone), group IV (IMQ+momethason), group V (IMQ+AP1%), group VI (IMQ+AP 0.175%), group VII (IMQ+ AP 0.25%).

**Fig. (8):**
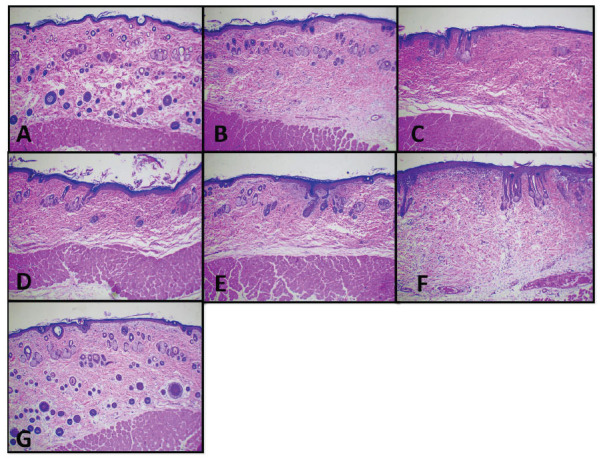
Histological examination results after seven days of treatment. **A**) Normal group (Group I), **B**) IMQ group (Group II), **C**) IMQ and Desoxymethasone (Group III), **D**) IMQ and Momethasone (Group IV), **E**) IMQ and 0.1% AP (Group V), **F**) IMQ and AP 0.175% (Group VI), and **G**) IMQ and AP 0.25% (Group VII).

**Table 1:** Binding affinity of ligands and receptors.

**Ligand**	**IL-6 (kcal/mol)**	**NF-kB (kcal/mol)**	**TNF-α (kcal/mol)**
Native Ligand	-5.65	-9.69	-4.32
Andrographolide	-5.92	-6.26	-6.18
Neoandrographolide	-6.43	-0.8	-6.87
Deoxyandrographolide	-6.06	-7.23	-6.47
Desoximetasone	-5.43	-9.53	-5.8
Hydrocortisone	-6.97	-8.18	-6.92
Mometasone	-6.5	-7.63	-6.33
Betametasone Dipropionate	-5.71	11.74	-7.1
Clobetasol Propionate	-5.26	-9.66	-6.01

**Table 2:** Binding affinity of ligands and receptors.

**Ligand**	**Conformation**	**Protein Target**	**Bond Energy (kcal/mol)**	**Amino Acid** **Residues**	**Hydrogen Bond** **Protein-ligand**
Native Ligand	-	IL-6	-5.65	ARG179 ARG179 ARG182 ARG182	HH21-O3 HE-O41 HH21-O41 HE-O4
	-	NF-KB	-9.69	PHE535	HN-O1
	-	TNF- α	-4.32	-	-
Andrographolide	2	IL-6	-5.92	ARG179 ARG179 ARG182	HE-O HH21-O HE-O
	7	NF-KB	-6.26	LEU472	HN-O
	9	TNF- α	-6.18	TYR151	HH-O
Neoandrographolide	2	IL-6	6.43	ARG179 ARG179 ARG182 GLN175	HE-O HH21-O HH21-O HE21-O
	1	NF-KB	-0.8	ARG408	HN-O
	-	TNF-α	-6.87	-	-
Deoxyandrographolide	1	IL-6	-6.06	GLN175	HE22-O
	-	NF-KB	-7.23	-	-
	2	TNF- α	-6.47	TYR151	HH-O
Desoximetasone	10	IL-6	-5.43	ARG182 ARG182	HE-O HH21-O
	3	NF-KB	-9.53	SER410	HN-O
	-	TNF-α	-5.8	-	-
Hydrocortisone	3	IL-6	-6.97	ARG179 ARG182 GLN175	HE-O HH21-O HE22-O
	2	NF-KB	-8.18	LEU472	HN-O
	7	TNF- α	-6.92	TYR151	HH-O
Mometasone	3	IL-6	-6.5	ARG179 ARG182	HE-O HH21-O
	7	NF-KB	-7.63	SER410	HN-O
	8	TNF- α	-6.33	TYR151	HH-O

**Table 3:** Correlation between toxicity profile variables and dimension of PCA.

**Toxicity Profile**	**Dim.1**	**Dim.2**
Water solubility	-0.8564509	-0.50169850
Caco^2^ permeability	0.0152015	0.86940900
Skin Permeability	0.4512152	-0.10158269
P-glycoprotein substrate	0.7535053	-0.46881943
P-glycoprotein I inhibitor	0.8952374	-0.09121417
P-glycoprotein II inhibitor	0.7523940	0.50826520
BBB permeability	-0.2135192	0.77089311
CNS permeability	0.3267130	0.48251502
Max. tolerated dose (human)	-0.9394901	0.21702161
Skin Sensitisation	NaN	NaN
*T.Pyriformis* toxicity	-0.4458822	0.79055851
Minnow toxicity	-0.3100385	-0.70566080

**Table 4:** Correlation between molecular interaction variables and dimension of PCA (continued).

**Molecular Interactions**	**Dim.1**	**Dim.2**
IL-6.tot	0.77637061	0.2789111
IL-6.vdW	0.89556174	-0.3730032
IL-6.EC	-0.73487852	0.6708242
NF-kB.tot	-0.77765708	-0.2667337
NF-kB.vdW	-0.86800665	-0.1630132
NF-kB.EC	0.07230658	0.9441718
TNF-α.tot	0.93222381	-0.2881415
TNF-α.vdW	0.49118632	0.6227135
TNF-α.EC	0.36591332	0.4264220

**Table 5:** Mean difference analysis of IL-6, IL-17, TNF-α, and VEGF among groups.

**Parameters**	**Groups**	**Mean ± SD**	***p*-Value**
IL-6	I	0.16 ± 0.23	0.242
-	II	6.83 ± 5.50	-
-	III	1.33 ± 0.33	-
-	IV	1.11 ± 0.38	-
-	V	1.55 ± 0.83	-
-	VI	2.33 ± 1.33	-
-	VII	1.55 ± 0.38	-
IL-17	I	0.16 ± 0.23	0.221
-	II	15.16 ± 12.16	-
-	III	2.67 ± 1.00	-
-	IV	1.55 ± 0.76	-
-	V	3.00 ± 1.45	-
-	VI	3.89 ± 3.56	-
-	VII	2.44 ± 1.07	-
TNF-α	I	0.33 ± 0.00	0.107
-	II	13.83 ± 10.16	-
-	III	3.11 ± 0.69	-
-	IV	2.66 ± 0.57	-
-	V	3.22 ± 1.07	-
-	VI	6.88 ± 3.15	-
-	VII	3.11 ± 1.07	-
VEGF	I	0.16 ± 0.23	0.201
-	II	6.00 ± 4.33	-
-	III	1.22 ± 0.50	-
-	IV	0.77 ± 0.50	-
-	V	1.11 ± 1.07	-
-	VI	2.11 ± 2.49	-
-	VII	1.11 ± 0.50	-

**Note:** Kruskal-Wallis analysis, Group I (normal), group II (IMQ), group III (IMQ+desoxymethasone), group IV (IMQ+momethason), group V (IMQ+AP 1%), group VI (IMQ+AP 0.175%), group VII (IMQ+ AP 0.25%).

**Table 6:** The parameters for observing the psoriasis progression.

**Parameters**	**Groups**	**Mean ± SD**	***p*-Value**
Final PASI Score	I	0.00 ± 0.00	0.017*
-	II	6.50 ± 2.121	-
-	III	2.33 ± 0.577	-
-	IV	1.67 ± 0.577	-
-	V	1.67 ± 0.577	-
-	VI	2.67 ± 0.577	-
-	VII	3.67 ± 0.577	-
Redness	I	0.00 ± 0.00	0.017*
-	II	2.50 ± 0.707	-
-	III	1.00 ± 1.33	-
-	IV	0.67 ± 0.577	-
-	V	1.00 ± 0.00	-
-	VI	1.00 ± 0.00	-
-	VII	1.00 ± 0.00	-
Scales	I	0.00 ± 0.00	0.040*
-	II	1.50 ± 0.707	-
-	III	0.33 ± 0.577	-
-	IV	0.00 ± 0.00	-
-	V	0.00 ± 0.00	-
-	VI	0.67 ± 0.577	-
-	VII	1.00 ± 0.00	-
Thickness	I	0.00 ± 0.00	0.023*
-	II	2.50 ± 0.707	-
-	III	1.00 ± 0.00	-
-	IV	1.00 ± 0.00	-
-	V	0.67 ± 0.577	-
-	VI	1.00 ± 0.00	-
-	VII	1.67 ± 0.577	-
Total histopathology of psoriasis score	I	1.00 ± 0.00	0.037*
	II	5.75 ± 4.59	-
-	III	2.16 ± 0.76	-
-	IV	1.67 ± 0.28	-
-	V	2.00 ± 0.00	-
-	VI	5.00 ± 2.29	-
-	VII	1.67 ± 0.57	-
Keratin layer score	I	0.50 ± 0.00	0.018*
-	II	2.50 ± 1.41	-
-	III	0.83 ± 0.57	-
-	IV	0.50 ± 0.00	-
-	V	0.50 ± 0.00	-
-	VI	2.16 ± 1.15	-
-	VII	0.50 ± 0.00	-
Epidermal layer score	I	0.00 ± 0.00	0.634
-	II	1.25 ± 1.76	-
-	III	0.50 ± 0.00	-
-	IV	0.33 ± 0.28	-
-	V	0.33 ± 0.28	-
-	VI	0.33 ± 0.28	-
-	VII	0.33 ± 0.28	-
Dermal layer score	I	0.50 ± 0.00	0.103
-	II	2.00 ± 1.41	-
-	III	0.83 ± 0.28	-
-	IV	0.83 ± 0.28	-
-	V	1.00 ± 0.00	-
-	VI	2.33 ± 1.15	-
-	VII	0.83 ± 0.28	-

**Note:** Kruskal-Wallis analysis, *p 0.05 (significant value). Group I (normal), group II (IMQ), group III (IMQ+desoxymethasone), group IV (IMQ+momethason), group V (IMQ+AP 1%), group VI (IMQ+AP 0.175%), group VII (IMQ+ AP 0.25%).

**Table 7:** Pairwise comparisons post hoc test (Dunn’s test) of final PASI score.

**Groups**		**Mean Difference**	**Std. Error**	***p*-value**
I	II	-17.000	5.454	0.002*
	III	-8.333	4.979	0.094
	IV	-5.000	4.979	0.315
	V	-5.000	4.979	0.315
	VI	-10.167	4.979	0.041*
	VII	-14.000	4.979	0.005*
II	I	-17.000	5.454	0.002*
	III	8.667	4.979	0.082
	IV	12.000	4.979	0.016*
	V	12.000	4.979	0.016*
	VI	6.833	4.979	0.170
	VII	3.000	4.979	0.547
III	I	-8.333	4.979	0.094
	II	8.667	4.979	0.082
	IV	3.333	4.453	0.016*
	V	3.333	4.453	0.454
	VI	-1.833	4.453	0.681
	VII	-5.667	4.453	0.203
IV	I	-5.000	4.979	0.315
	II	12.000	4.979	0.016*
	III	3.333	4.453	0.016*
	V	0.000	4.453	1.000
	VI	-5.167	4.453	0.246
	VII	-9.000	4.453	0.043*
V	I	-5.000	4.979	0.315
	II	12.000	4.979	0.016*
	III	3.333	4.453	0.454
	IV	0.000	4.453	1.000
	VI	-5.167	4.453	0.246
	VII	-9.000	4.453	0.043*
VI	I	-10.167	4.979	0.041*
	II	6.833	4.979	0.170
	III	-1.833	4.453	0.681
	IV	-5.167	4.453	0.246
	V	-5.167	4.453	0.246
	VII	-3.833	4.453	0.389
VII	I	-14.000	4.979	0.005*
	II	3.000	4.979	0.547
	III	-5.667	4.453	0.203
	IV	-9.000	4.453	0.043*
	V	-9.000	4.453	0.043*
	VI	-3.833	4.453	0.389

**Note:** Dunn’s test analysis, *p 0.05 (significant value). Group I (normal), group II (IMQ), group III (IMQ+desoxymethasone), group IV (IMQ+momethason), group V (IMQ+AP 1%), group VI (IMQ+AP 0.175%), group VII (IMQ+ AP 0.25%).

**Table 8:** Pairwise comparisons post hoc test (Dunn’s test) of scale score.

**Groups**		**Mean Difference**	**Std. Error**	***p*-value**
I	II	-11.000	4.899	0.025*
	III	-3.000	4.472	0.502
	IV	0.000	4.472	1.000
	V	0.000	4.472	1.000
	VI	-6.000	4.472	0.180
	VII	-9.000	4.472	0.044*
II	I	-11.000	4.899	0.025*
	III	8.000	4.472	0.074
	IV	11.000	4.472	0.014*
	V	11.000	4.472	0.014*
	VI	5.000	4.472	0.264
	VII	2.000	4.472	0.655
III	I	-3.000	4.472	0.502
	II	8.000	4.472	0.074
	IV	3.000	4.000	0.453
	V	3.000	4.000	0.453
	VI	-3.000	4.000	0.453
	VII	-6.000	4.000	0.134
IV	I	0.000	4.472	1.000
	II	11.000	4.472	0.014*
	III	3.000	4.000	0.453
	V	0.000	4.000	1.000
	VI	-6.000	4.000	0.134
	VII	-9.000	4.000	0.024*
V	I	0.000	4.472	1.000
	II	11.000	4.472	0.014*
	III	3.000	4.000	0.453
	IV	0.000	4.000	1.000
	VI	-6.000	4.000	0.134
	VII	-9.000	4.000	0.024*
VI	I	-6.000	4.472	0.180
	II	5.000	4.472	0.264
	III	-3.000	4.000	0.453
	IV	-6.000	4.000	0.134
	V	-6.000	4.000	0.134
	VII	-3.000	4.000	0.453
VII	I	-9.000	4.472	0.044
	II	2.000	4.472	0.655
	III	-6.000	4.000	0.134
	IV	-9.000	4.000	0.024*
	V	-9.000	4.000	0.024*
	VI	-3.000	4.000	0.453

**Note:** Dunn’s test analysis, *p 0.05 (significant value). Group I (normal), group II (IMQ), group III (IMQ+desoxymethasone), group IV (IMQ+momethason), group V (IMQ+AP 1%), group VI (IMQ+AP 0.175%), group VII (IMQ+ AP 0.25%).

**Table 9:** Pairwise comparisons post hoc test (Dunn’s test) of thickness score.

**Groups**		**Mean Difference**	**Std. Error**	***p*-value**
I	II	-16.000	4.848	0.001*
	III	-7.500	4.425	0.090
	IV	-7.500	4.425	0.090
	V	-5.000	4.425	0.259
	VI	-7.500	4.425	0.090
	VII	-12.500	4.425	0.005*
II	I	-16.000	4.848	0.001
	III	0.000	3.958	1.000
	IV	8.500	4.425	0.055
	V	11.000	4.425	0.013*
	VI	8.500	4.425	0.055
	VII	3.500	4.425	0.429
III	I	-7.500	4.425	0.090
	II	8.500	4.425	0.055
	IV	0.000	3.958	1.000
	V	2.500	3.958	0.528
	VI	0.000	3.958	1.000
	VII	-5.000	3.958	0.207
IV	I	-7.500	4.425	0.090
	II	8.500	4.425	0.055
	III	0.000	3.958	1.000
	V	2.500	3.958	0.528
	VI	0.000	3.958	1.000
	VII	-5.000	3.958	0.207
V	I	-5.000	4.425	0.259
	II	11.000	4.425	0.013*
	III	2.500	3.958	0.528
	IV	2.500	3.958	0.528
	VI	-2.500	3.958	0.528
	VII	-7.500	3.958	0.058
VI	I	-7.500	4.425	0.090
	II	8.500	4.425	0.055
	III	0.000	3.958	1.000
	IV	0.000	3.958	1.000
	V	-2.500	3.958	0.528
	VII	-5.000	3.958	0.207
VII	I	-12.500	4.425	0.005*
	II	3.500	4.425	0.429
	III	-5.000	3.958	0.207
	IV	-5.000	3.958	0.207
	V	-7.500	3.958	0.058*
	VI	-5.000	3.958	0.207

**Note:** Dunn’s test analysis, *p 0.05 (significant value). Group I (normal), group II (IMQ), group III (IMQ+desoxymethasone), group IV (IMQ+momethason), group V (IMQ+AP 1%), group VI (IMQ+AP 0.175%), group VII (IMQ+ AP 0.25%).

**Table 10:** Pairwise comparisons post hoc test (Dunn’s test) of redness score.

**Groups**		**Mean Difference**	**Std. Error**	***p*-value**
I	II	-16.500	4.349	0.000*
	III	-8.500	3.970	0.032*
	IV	-5.667	3.970	0.154
	V	-8.500	3.970	0.032*
	VI	-8.500	3.970	0.032*
	VII	-8.500	3.970	0.032*
II	I	-16.500	4.349	0.000*
	III	8.000	3.970	0.044*
	IV	10.833	3.970	0.006*
	V	8.000	3.970	0.044*
	VI	8.000	3.970	0.044*
	VII	8.000	3.970	0.044*
III	I	-8.500	3.970	0.032*
	II	8.000	3.970	0.044*
	IV	2.833	3.551	0.425*
	V	0.000	3.551	1.000
	VI	0.000	3.551	1.000
	VII	0.000	3.551	1.000
IV	I	-5.667	3.970	0.154
	II	10.833	3.970	0.006*
	III	2.833	3.551	0.425
	V	-2.833	3.551	0.425
	VI	-2.833	3.551	0.425
	VII	-2.833	3.551	0.425
V	I	-8.500	3.970	0.032*
	II	8.000	3.970	0.044*
	III	0.000	3.551	1.000
	IV	0.000	3.551	1.000
	VI	0.000	3.551	1.000
	VII	0.000	3.551	1.000
VI	I	-8.500	3.970	0.032*
	II	8.000	3.970	0.044*
	III	0.000	3.551	1.000
	IV	-2.833	3.551	0.425
	V	0.000	3.551	1.000
	VII	0.000	3.551	1.000
VII	I	-8.500	3.970	0.032*
	II	8.000	3.970	0.044*
	III	0.000	3.551	1.000
	IV	-2.833	3.551	0.425
	V	0.000	3.551	1.000
	VI	0.000	3.551	1.000

**Note:** Dunn’s test analysis, *p 0.05 (significant value). Group I (normal), group II (IMQ), group III (IMQ+desoxymethasone), group IV (IMQ+momethason), group V (IMQ+AP 1%), group VI (IMQ+AP 0.175%), group VII (IMQ+ AP 0.25%).

**Table 11:** Pairwise comparisons post hoc test (Dunn’s test) of psoriasis score according to histology examination.

**Groups**		**Mean Difference**	**Std. Error**	***p*-value**
I	II	-4.667	4.988	0.350
	III	-5.333	4.988	0.285
	IV	-8.000	4.988	0.109
	V	-8.333	4.988	0.095
	VI	-14.500	4.988	0.004*
	VII	-14.650	5.465	0.007*
II	I	-4.667	4.988	0.350
	III	6.417	4.988	0.198
	IV	10.083	4.988	0.043*
	V	6.750	4.988	0.176
	VI	0.250	4.988	0.960
	VII	9.417	4.988	0.059
III	I	-5.333	4.988	0.285
	II	6.417	4.988	0.198
	IV	3.667	4.462	0.455
	V	0.333	4.462	0.940
	VI	-6.167	4.462	0.167
	VII	3.000	4.462	0.501
IV	I	-8.000	4.988	0.109
	II	10.083	4.988	0.043*
	III	3.667	4.462	0.411
	V	-3.333	4.462	0.881
	VI	-9.833	4.462	0.028*
	VII	-0.667	4.462	0.455
V	I	-8.333	4.988	0.095
	II	6.750	4.988	0.176
	III	0.333	4.462	0.940
	IV	-3.333	4.462	0.881
	VI	-6.500	4.462	0.145
	VII	2.667	4.462	0.550
VI	I	-14.500	4.988	0.004*
	II	0.250	4.988	0.960
	III	-6.167	4.462	0.167
	IV	-9.833	4.462	0.028*
	V	-6.500	4.462	0.145
	VII	9.167	4.462	0.040*
VII	I	-14.650	5.465	0.007*
	II	9.417	4.988	0.059
	III	3.000	4.462	0.501
	IV	-0.667	4.462	0.455
	V	2.667	4.462	0.550
	VI	9.167	4.462	0.040*

**Note:** Dunn’s test analysis, *p 0.05 (significant value). Group I (normal), group II (IMQ), group III (IMQ+desoxymethasone), group IV (IMQ+momethason), group V (IMQ+AP 1%), group VI (IMQ+AP 0.175%), group VII (IMQ+ AP 0.25%).

**Table 12:** Pairwise comparisons post hoc test (Dunn’s test) of keratin score.

**Groups**		**Mean Difference**	**Std. Error**	***p*-value**
I	II	-10.000	4.610	0.030*
	III	-2.833	4.208	0.501
	IV	0.000	4.208	1.000
	V	0.000	4.208	1.000
	VI	-9.500	4.208	0.024*
	VII	0.000	4.208	1.000
II	I	-10.000	4.610	0.030*
	III	7.167	4.208	0.089
	IV	10.00	4.208	0.017*
	V	10.000	4.208	0.017
	VI	0.500	4.208	0.905
	VII	10.000	4.208	0.017*
III	I	-2.833	4.208	0.501
	II	7.167	4.208	0.089
	IV	0.000	4.208	1.000
	V	0.000	4.208	1.000
	VI	-6.667	3.764	0.077
	VII	2.833	3.746	0.452
IV	I	0.000	4.208	1.000
	II	10.00	4.208	0.017*
	III	2.833	3.746	0.452
	V	0.000	3.746	1.000
	VI	-9.500	3.746	0.012*
	VII	0.000	3.746	1.000
V	I	0.000	4.208	1.000
	II	10.000	4.208	0.017*
	III	2.833	3.674	0.452
	IV	0.000	3.746	1.000
	VI	-9.500	3.674	0.012
	VII	0.000	3.674	1.000
VI	I	-9.500	4.208	0.024*
	II	0.500	4.208	0.905
	III	-6.667	3.764	0.077
	IV	-9.500	3.746	0.012*
	V	-9.500	3.674	0.012
	VII	9.500	3.746	0.012
VII	I	0.000	4.208	1.000
	II	10.000	4.208	0.017
	III	2.833	3.746	0.452
	IV	0.000	3.746	1.000
	V	0.000	3.674	1.000
	VI	9.500	3.746	0.012

**Note:** Dunn’s test analysis, *p 0.05 (significant value). Group I (normal), group II (IMQ), group III (IMQ+desoxymethasone), group IV (IMQ+momethason), group V (IMQ+AP 1%), group VI (IMQ+AP 0.175%), group VII (IMQ+ AP 0.25%).

## Data Availability

The data and supportive information are available within the article.

## LIST OF ABBREVIATIONS

AP: Andrographolide
DAP: Deoxyandrographolide
DC: Dendritic Cells
ESAF: Endothelial Cell Stimulating Angiogenesis Factor
IL: Interleukin
NAP: Neoandrographolide
PASI: Psoriasis Area And Severity Index
TNF-α: Tumor Necrosis Factor-alpha
